# Natural CCD2 Variants and RNA Interference for Boosting Crocin Biosynthesis in Tomato

**DOI:** 10.3390/biology14070850

**Published:** 2025-07-12

**Authors:** Elena Moreno-Giménez, Eduardo Parreño, Lucía Morote, Alberto José López Jiménez, Cristian Martínez Fajardo, Silvia Presa, Ángela Rubio-Moraga, Antonio Granell, Oussama Ahrazem, Lourdes Gómez-Gómez

**Affiliations:** 1 Instituto Botánico, Departamento de Ciencia y Tecnología Agroforestal y Genética, Universidad de Castilla-La Mancha, Campus Universitario s/n, 02071 Albacete, Spain; elena.morenogimenez@uclm.es (E.M.-G.); eduardo.parreno@uclm.es (E.P.); albertojose.lopez@uclm.es (A.J.L.J.); cristian.martinez@uclm.es (C.M.F.); angela.rubio@uclm.es (Á.R.-M.);; 2 Instituto de Biología Molecular y Celular de Plantas, Consejo Superior de Investigaciones Científicas-Universidad Politécnica de València, 46022 Valencia, Spain; silviapr@upvnet.upv.es (S.P.); agranell@ibmcp.upv.es (A.G.)

**Keywords:** Crocosmia, saffron, tomato, crocins, carotenoid cleavage dioxygenase, glucosyltransferases, violaxanthin synthase

## Abstract

Crocin biosynthesis involves a complex network of enzymes with biosynthetic and modifier enzymes, and the manipulation of these pathways holds promise for improving human health through the broad exploitation of these bioactive metabolites. Crocins play a significant role in human nutrition and health, as they exhibit antioxidant and anti-inflammatory activity. Plants that naturally accumulate high levels of crocins are scarce, and the production of crocins is highly limited by the characteristics of the crops and their yield. The CCD2 enzyme, initially identified in saffron, is responsible for converting zeaxanthin into crocetin, which is further modified to crocins by aldehyde dehydrogenases and glucosyltransferase enzymes. Crops like tomato fruits, which naturally contain high levels of carotenoids, offer valuable genetic resources for expanding synthetic biology tools. In an effort to explore CCD2 enzymes with improved activity, two CCD2 alleles from saffron and Crocosmia were introduced into tomato, together with a UGT gene. Furthermore, in order to increase the zeaxanthin pool in the fruit, an RNA interference construct was introduced to limit the conversion of zeaxanthin to violaxanthin. The expression of saffron CCD2, CsCCDD2L, led to the creation of transgenic tomatoes with significantly high crocins levels, reaching concentrations of 4.7 mg/g dry weight. The Crocosmia allele, CroCCD2, also resulted in high crocins levels, reaching a concentration of 2.1 mg/g dry weight. These findings underscore the importance of enzyme variants in synthetic biology, as they enable the development of crops rich in beneficial apocarotenoids.

## Simple Summary

This study addresses the challenge of producing crocins, which are natural compounds with strong antioxidant and anti-inflammatory benefits for human health. Currently, few plants make crocins in large amounts, making them hard to grow in large quantities. The goal of this research was to use genetic engineering to create tomatoes that can produce high levels of crocins, offering a new, sustainable source of these valuable nutrients. Spe-cific genes from saffron and a plant called Crocosmia were introduced into tomatoes, along with a gene to boost the production of crocins by increasing the levels of zeaxanthin, a natural pigment needed for their production. As a result, the genetically modified toma-toes produced crocins at significantly high levels. This study shows that selecting the right versions of key enzymes can greatly improve efficiency in producing health-boosting compounds in everyday crops.

## 1. Introduction

**Figure 1:**
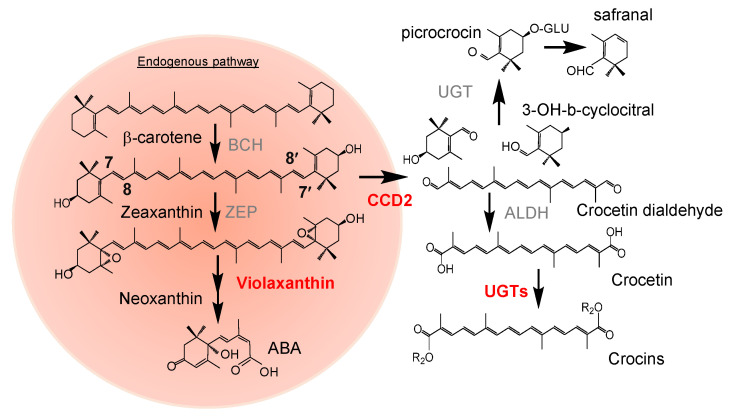
A schematic representation of the part of the carotenoid pathway from β-carotene to ABA, included on the red background, and on the right part of the figure is shown the crocin biosynthetic pathway elucidated in saffron. BCH, β-carotene hydroxilase; ZEP, zeaxanthin epoxidase; CCD2; carotenoid cleavage dioxygenase 2; ABA, abscisic acid; ALDH, aldehyde dehydrogenase; UGT, UDP-glucosyltransferase.

Pigmentation holds crucial ecological significance in living organisms, serving as a vital mediator between them and their surrounding environment. The industrial value of plant pigments encompasses multiple fields, including food, agriculture, cosmetics, pharmaceuticals, and chemistry. Plant pigmentation consists of a diverse array of secondary metabolites, including anthocyanins, betalains, and carotenoids [1]. Carotenoids are among the most commonly found pigments in nature, producing yellow, orange, and red hues. Their typical structure includes a polyene chain with conjugated double bonds, which are susceptible to cleavage by the enzymatic activity of carotenoid cleavage dioxygenases [CCD], producing apocarotenoids. These compounds could subsequently undergo a broad range of modifications to yield biologically active molecules, playing diverse roles across all living organisms [2,3]. Certain apocarotenoids show attractive colors due to their strong absorption in the visible spectrum as in the case of bixin, heteranthin, ditaxin, β-citraurin, crocetin and crocins. Crocin and picrocrocin are water-soluble apocarotenoids found in many *Crocus* species [4,5] and accumulate in significant amounts in saffron [*Crocus sativus*] and in smaller quantities in Buddleja [6]. In addition to saffron, crocins are also obtained from the fruits of gardenia [7]. Besides their pigment properties, crocins are also known for their therapeutic potential due to their antioxidant and anti-inflammatory properties [8], while picrocrocin, the percussor of the odor-active volatile safranal, also has also shown different medicinal properties [9]. In monocotyledonous species, crocins and picrocrocin are derived from the cleavage of the carotenoid zeaxanthin at the 7,8:7′,8′ positions through a reaction catalyzed by carotenoid cleavage dioxygenase enzymes that belong to the CCD2 group. CCD2 enzymes have been identified in a restricted number of species, including species from the genus *Crocus* [5,10,11], *Freesia* [12], and *Crocosmia* × *crocosmiiflora* [13]. This reaction generates two molecules of 6,6-trimethyl-4-hydroxy-1-carboxaldehyde-1-cyclohexene [HTCC] and one molecule of crocetin dialdehyde, becoming water-soluble through the action of glucosyltransferases [UGT], which have been found to catalyze the addition of glucose molecules [14,15,16] [Figure 1].

To date, the stigma of saffron is the main source of crocins and picrocrocin. To obtain one kilogram of the spice, approximately 250,000 stigmas must be collected. These are obtained through the manual dissection of flowers, which are also harvested manually in the field only once a year over a relatively short period of time. This highly labor-intensive process, which is also heavily dependent on climatic conditions, is the main constraint that determines the high value of the spice [17]. The enzymes from saffron and Crocosmia haven been transiently produced in *Nicotiana benthamiana* plants using a viral vector, allowing the generation of 6.63 ± 1.28 mg and 4.37 ± 0.07 mg of crocins per gram of *N. benthamiana* dry weight [DW] leaf tissue, respectively [13]. However, stable transformation systems to produce secondary metabolites are desirable in terms of yield optimization, cost reduction, and ease of handling, as well as requiring fewer infrastructure resources and less specialized personnel for continuous management. During the last few years, research with the CCD2 enzyme from saffron [CsCCD2] has demonstrated the ability to synthesize crocins in tomato fruits [18,19], where the major limitation is the zeaxanthin content in the fruit. A strategy to enhance zeaxanthin content was established based on the introduction of CsCCD2 into a tomato mutant carrying the *high-pigment 3* [*hp3*] mutation [20] and the B^Sh^ gene, corresponding to the genomic DNA sequence of a chromoplast-specific lycopene β-cyclase, from *Solanum habrochaites* [21], which exhibits increased accumulation of β-carotene and zeaxanthin in the mature fruit [22]. Using this strategy, it was possible to obtain crocin levels close to 6.5 mg/g DW [19]. Previous studies with *hp3* plants showed increased levels of zeaxanthin, reaching values above 5.1 µg/g FW compared with the 7.7 µg/g FW obtained with the combinations of *hp3* and B^Sh^ plants [22]. The *hp3* mutation affected the zeaxanthin epoxidase [ZEP] enzyme, which converts zeaxanthin to violaxanthin. Consequently, abscisic acid levels [ABA] are strongly reduced in all of the tissues of this plant, and as a result, field-grown plants exhibit increased susceptibility to water stress, reduced overall biomass and yield, and accelerated leaf water loss, characteristics that are undesirable when aiming to develop an efficient biofactory for the cost-effective production of high-value metabolites. To overcome this challenge, we selectively suppressed the expression of *ZEP* in tomato fruit through RNA interference and simultaneously expressed *CsCCD2L* or *CroCCD2* to achieve the production and accumulation of the valuable apocarotenoids crocin and picrocrocin. Further, the constructs were completed with the introduction of the *UGT91P3* gene, which encodes a UGT involved in the sugar chain elongation of crocins [16].

## 2. Materials and Methods

### 2.1. Strucltural Charcaterization of CsCCDD2L and CroCCD2

Protein three-dimensional [3D] structures were visualized and analyzed using UCSF Chimera [version 1.19], a molecular modeling program. Protein models were generated by homology modeling for proteins lacking experimental structures using the Phyre 2.0 server. The structures were imported into Chimera, and missing residues or loops were modeled when necessary to ensure completeness. To assess the distribution of hydrophobic and hydrophilic regions, surface hydrophobicity mapping was performed using the Kyte-Doolittle (https://web.expasy.org/protscale/, (accessed on 6 July 2025)) scale integrated into Chimera. The protein surface was colored based on hydrophobicity values, with hydrophobic residues highlighted in warmer colors [orange to red] and hydrophilic residues in cooler colors [blue], allowing the identification of potential ligand-binding sites or interaction interfaces. All structural images and hydrophobicity maps were generated within Chimera and exported in high resolution for presentation.

### 2.2. Generation of MMO16 and MMO17 Tomato Plants

Constructs were generated using the GoldenBraid 4.0 platform [https://gbcloning.upv.es/, accessed on 6 July 2025]. For the ZEP-RNAi construct, a 1923 bp fragment of the ZEP cDNA sequence [locus NM_001309304] was selected, starting from position 41 relative to the 5′ end. The RNAi fragment was amplified via PCR using Advantage DNA polymerase [Takara, Tokyo, Japan] and tomato cDNA as a template, with the primers ZEP-RNAi-F and ZEP-RNAi-R [Table S1] designed according to the GoldenBraid protocol. The amplified fragment was purified from an agarose gel and cloned into the level 0 vector pUPD2 following the GoldenBraid guidelines. The ZEP-RNAi construct, along with the CsCCD2L, CsUGTgg, and CroCCD2 genes, previously amplified from saffron and Crocosmia cDNA [Table S1], were assembled into modular cloning vectors using the GoldenBraid system. The resulting binary vectors were O16: pDGBα1R pE8-CroCCD2-T35S:p35S-CsUGTgg-T35S:p2A11-Zepox-T35S:pNos-nptII-TNos and O17: pDGBα1R pE8-CsCCD2-T35S:p35S-CsUGTgg-T35S:p2A11-Zepox-T35S:pNos-nptII-TNos.

The *Agrobacterium tumefaciens*-mediated transformation of tomato cv. Money Maker was carried out as previously described [23]. Transgenic plants were confirmed by kanamycin resistance and PCR amplification using promoter- and gene-specific primers [Table S1]. Successfully transformed plants were transferred to soil and cultivated in a greenhouse under controlled conditions.

### 2.3. Extracts of Tomato Fruit

Fruits were harvested ten days post breaker, with at least three independent biological replicates. Fruits were cut in half, and seeds were removed before being frozen in liquid nitrogen and lyophilized, followed by storage at −80 °C. For the obtention of juices, fresh fruits were processed as previously described [18].

### 2.4. Gene Expression Analysis

RNA was extracted from fruit with Trizol Reagent, according to the manufacturer’s protocol [Thermo, Waltham, MA, USA]. DNaseI-treated RNA samples [Thermo, Waltham, MA, USA] were reverse transcribed using a cDNA reverse transcription kit [Thermo, Waltham, MA, USA]. Gene expression levels were analyzed using SYBR^®^ Green [Promega, Madison, WI, USA] and ABI PRISM™ StepOne sequence Detection System [Applied Biosystems, Waltham, MA, USA] PCR instruments. The primers used are listed in Table S1. Actin was used as an endogenous reference gene.

### 2.5. Carotenoid and Apocarotenoid Extraction and Analysis

Carotenoids and polar apocarotenoids were extracted from 50 mg of lyophilized tomato pericarp. Tissue prep, pigment extraction, and further analysis were carried out as described previously [13]. All solvents used were HPLC grade.

### 2.6. Statistical Analysis

Data are presented as the mean ± standard deviation. The significance of differences between the transgenic lines and WT plants was analyzed using Student’s *t*-test [** *p* < 0.05, *** *p* < 0.01].

## 3. Results

### 3.1. Two Constructs for Ectopic Expression of Saffron and Crocosmia CCD2 Enzymes and Suppressed ZEP Expression in Tomato Transgenic Lines

**Figure 2:**
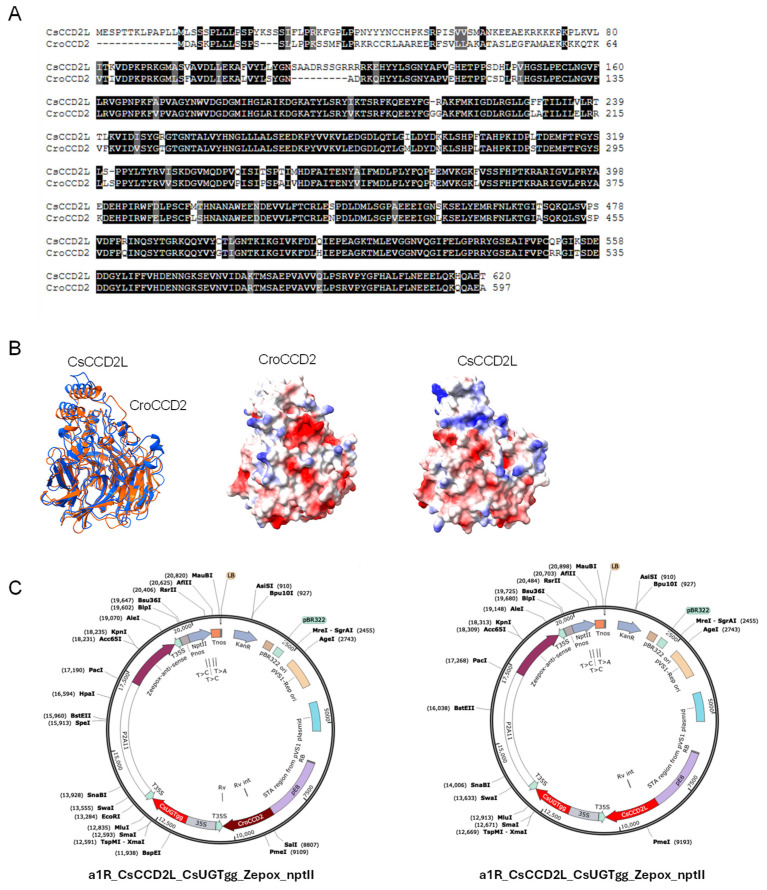
Comparison analyses between CroCCd2 and CsCCD2L and constructs used for tomato transformation. (**A**) Alignment of primary sequences. (**B**) Tridimensional structure and electrostatic surfaces of molecular models of CroCCD2 (orange) and CsCCD2L (blue). Blue indicates positively charged residues, red represents negative areas, and white represents neutral regions. (**C**) Vector maps of the two constructs stably transformed in tomato cv. Monkey Maker plants.

CsCCD2L and CroCCD2 showed 78.38% identity at the amino acid level [Figure 2A], with major differences found in the N-t region, more specifically in the sequences encoding the signal for targeting plastids. However, other differences were found as well throughout the entire sequences [Figure 2A]. The tridimensional structural models of both proteins were built and compared [Figure 2B]. The docking results indicated that the presence of different amino acids between both enzymes affected the flexibility of the proteins but had minimal effects on the typical structure of the enzymes. The specific arrangement of β-strands and α-helices showed a conserved core structure characteristic of CCDs [24]. The electrostatic surfaces of both proteins are shown in Figure 2B, suggesting that the amino acid differences have a high impact on the electrostatic surface when comparing both enzymes.

**Figure 3:**
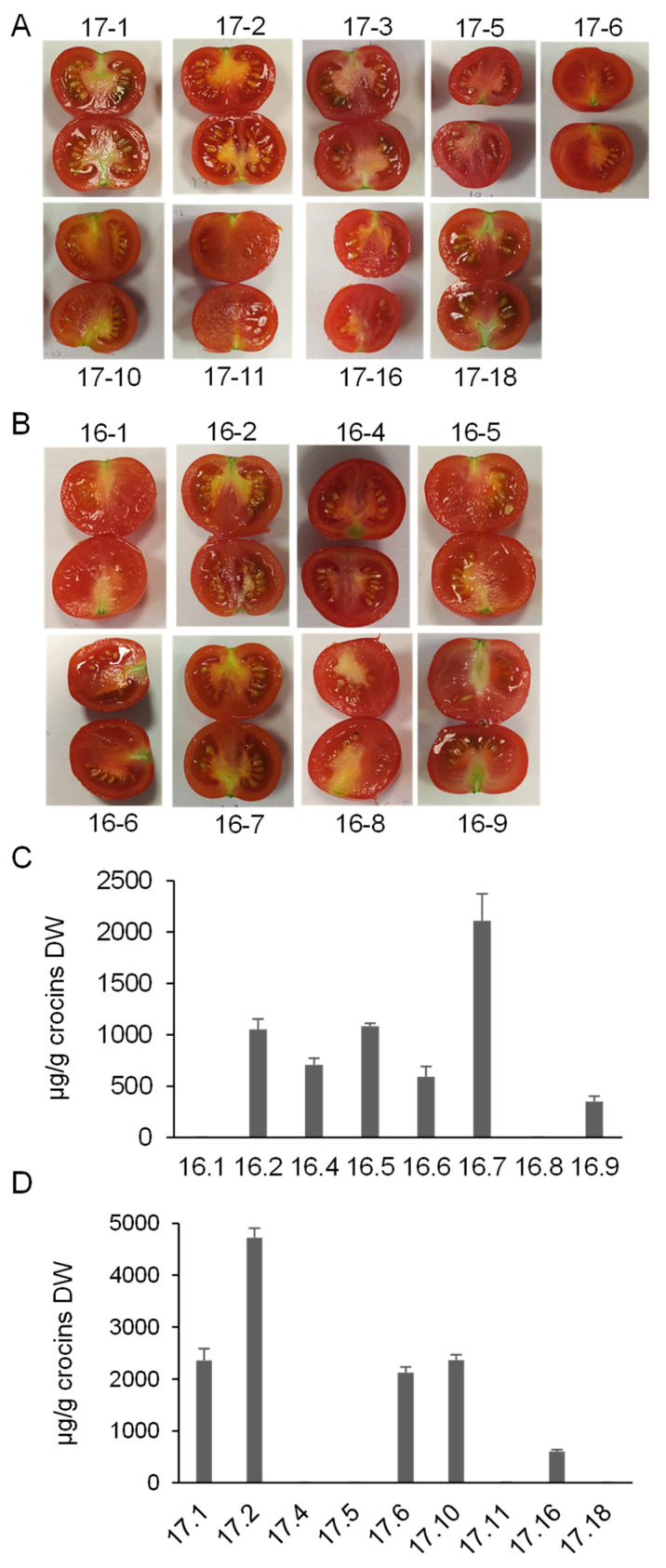
Crocin accumulation in tomato fruits from the different transgenic lines obtained. (**A**) Representative tomato fruits from the MMO16 lines expressing CroCCD2. (**B**) Representative tomato fruits from the MMO17 lines expressing CsCCD2L. (**C**) Crocins levels in the MMO16 lines. (**D**) Crocins levels in the MMO17 lines.

To test how the differences at the sequence level can impact the activity of these enzymes, both were assayed for crocin production in tomato fruits. Since previous studies on transgenic tobacco plants constitutively expressing *CsCCD2L* showed that the problems associated with the activity of this enzyme translate to bleaching phenotypes and poor crocins levels [25,26], we used a fruit-specific promoter to avoid undesirable effects on tomato plant development. The *CsCCD2L* and *CroCCD2* genes from saffron and Crocosmia, respectively, were independently cloned into an expression cassette containing the E8 promoter from tomato and the 35S terminator from CaMV 35S. Then, to further increase crocin biosynthesis, *UGT91P3* gene expression was driven by the constitutive CaMV 35S and assembled in the same construct as *CsCCD2L* or *CroCCD2.* In addition, to enhance the accumulation of zeaxanthin in the carotenoid pathway, *ZEP* expression was suppressed via RNA interference driven by the 2A11 promoter and containing a cDNA targeting the gene-specific coding region of ZEP. Ten stably transformed tomato lines using *A. tumefaciens* were established for each construct and named MMO16 [CroCCD2-CsUGTgg-Zepox-ntpII] and MMO17 [CsCCD2L-CsUGTgg-Zepox-ntpII] [Figure 2C]. Of the ten plants obtained for MMO16, eight produced fruits, while of the ten plants obtained for MMO17, only one plant did not produce fruits. Visually, the fruits of all lines showed a red phenotype, but inside the fruits an orange coloration was observed in several lines at the level of placenta [Figure 3A].

### 3.2. Evaluation of Crocin Levels in the Transgenic Tomato Lines

Fruits from the 17 developed plants [Figure 3A,B] were analyzed ten days post breaker [Br + 10] for crocin accumulation and gene expression levels for the *CCD2* genes, *UGT91P3*, and *ZEP* by quantitative Reverse Transcription PCR [qRT-PCR].

We freeze-dried the fruit tissue to estimate crocin levels on the basis of dry weight. Six out of the nine MMO17 lines were positive for crocin accumulation, with very variable levels [Figure 3C]. Line MMO17.2 showed the highest levels [Figure 3C and Figure S1], followed by the lines MMO17.1, MMO17.6, and MMO17.10, which showed higher levels of crocins compared with the lines MMO17.16 and MMO17.11 [Figure 3C]. Among the MMO16 lines, the line MMO16.7 showed the highest crocin content [Figure 3B and Figure S1], and two lines, MMO16.1 and MMO16.8, were negative for crocin accumulation [Figure 3D].

**Figure 4:**
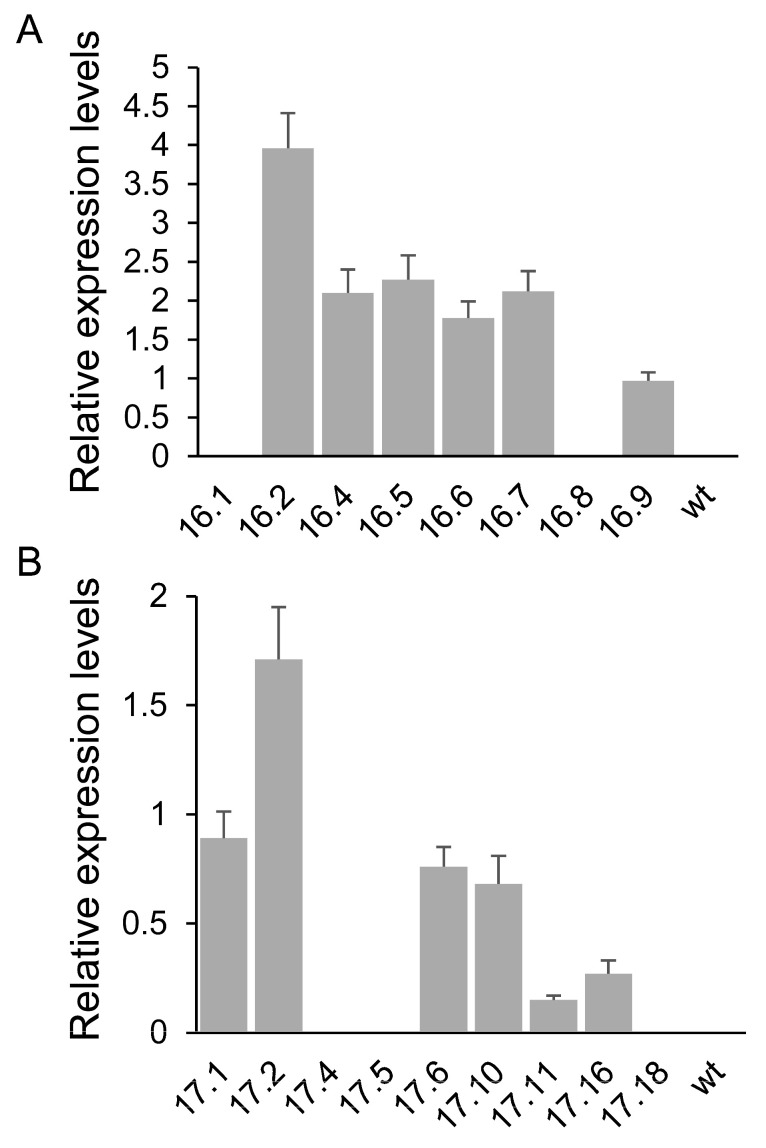
qRT-PCR expression analysis of the two CCD2 genes in transgenic lines and WT plants. Total RNA from the pericarp tissues of fruits collected 10 days after breaker was subjected to quantitative RT-PCR analysis. (**A**) Transcript levels of *CroCCD2*. (**B**) Transcript levels of *CsCCD2L*.

We completed this analysis by assessing the expression of *CroCCD2* and *CsCCD2L* in the transgenic lines MMO16 and MMO17, respectively. Transformed lines expressed the *CroCCD2* transgene at different levels; however, the lines MMO16.1 and MMO16.8 showed no expression of *CroCCD2* [Figure 4A]. All the MMO17-transformed lines positive for crocin accumulation expressed the *CsCCD2L* transgene [Figure 4B], while it was not detected in the lines 17.4, 17.5, and 17.18.

### 3.3. Carotenoid Levels in the Transgenic Tomato Lines

**Figure 5:**
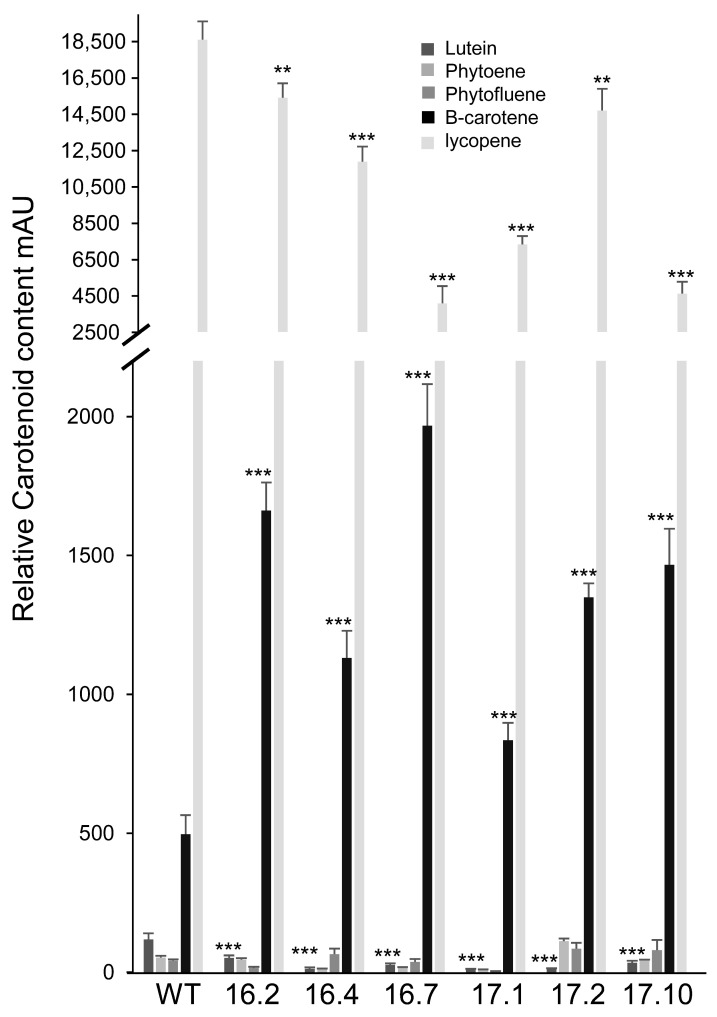
Carotenoid levels in selected transgenic and WT tomato fruits collected 10 days after breaker. Phytoene was detected at 250 nm, phytofluene was detected at 350 nm, lutein and β-carotene were detected at 450 nm, and lycopene was detected at 500 nm. ** *p* < 0.05 *** *p* < 0.01.

Carotenoids were analyzed in lines that showed a higher content of crocins (MMO17.1, MMO17.2, MMO17.6, MMO17.10, MMO 16.2, MM16.4, and MMO16.7) and compared to WT plants [Figure 5]. Lycopene was the most abundant carotenoid across all samples. Compared to the WT plants, all lines exhibited a reduction in both lycopene and lutein levels. Notably, β-carotene levels were elevated in all MMO16 and MMO17 lines relative to the WT plants [Figure 5]. This increase in β-carotene may be attributed to the downregulation of the ε,β-carotene branch of the carotenoid biosynthesis pathway, evidenced by the reduced lutein content, which likely redirected metabolic flux toward the β,β-carotene branch, thereby enhancing β-carotene accumulation.

## 4. Discussion

Lifestyle choices significantly influence both inflammation and oxidative stress in the body, two interconnected processes that can impact overall health. For instance, oxidative stress can activate inflammatory pathways, while chronic inflammation can lead to the increased production of free radicals, perpetuating a cycle that contributes to various diseases such as neurodegenerative disorders like Parkinson’s and Alzheimer’s. The growing impact of deaths and disabilities linked to neurological disorders is increasingly acknowledged as a major global public health concern, with projections indicating a further rise in the coming decades due to the transition toward an increasingly aging population [27]. Consequently, the prevention and management of these conditions have emerged as global health priorities, with the absence of effective treatments underscoring the urgency of developing preventive strategies [28]. Crocin, a bioactive carotenoid predominantly found in saffron, has been extensively investigated for its potential therapeutic effects. Evidence from both clinical and preclinical studies indicates that crocin administration may serve as an effective adjunct in the management of neurobiological disorders, primarily owing to its strong antioxidant and anti-inflammatory properties [29,30]. Since dietary interventions are generally preferred over pharmacological treatments in the early stages of neurodegenerative diseases, the inclusion of neuroprotective compounds in daily nutrition could play a crucial role in slowing disease progression and alleviating symptoms [31]. Saffron represents the most extensively studied natural source of crocin, and its integration into various food matrices—including baked goods, beverages, dairy products, desserts, and cereals—has been demonstrated to enhance sensory properties, extend shelf life, and increase antioxidant activity. Additionally, its incorporation may contribute to disease risk reduction and the promotion of overall health [32]. Thus, the regular intake of crocin-enriched foods could serve as a promising approach for preventing neurodegenerative diseases. However, the large-scale production of saffron presents considerable challenges, spanning cultivation, post-harvest processing, and final applications to obtain adequate amounts of bioactive compounds [33].

To expand the repertoire of plant species capable of accumulating crocin, tomato was selected for crocin biosynthesis and accumulation [18,19]. This platform can be further optimized by enhancing carotenoid precursor accumulation, such as by increasing zeaxanthin levels using different species or through metabolic engineering [22,34,35], or by restricting its downstream conversion to violaxanthin. Additionally, by selectively targeting these metabolic modifications to the fruit, potential disruptions in vegetative tissues can be minimized.

We specifically inhibited ZEP expression in tomato fruits and at the same time expressed two different CCD2 enzymes from *C. sativus* and *Crocosmia* × *crocosmiiflora*. For this purpose, we used the GoldenBraid 2.0 strategy, which allows multigene assembly. We assembled and expressed three genes simultaneously in tomato fruit. These genes were regulated by the fruit-specific promoters pE8 and p2A11, aiming to enhance the levels of zeaxanthin for crocins biosynthesis.

Variation in crocin accumulation among independent transgenic lines was observed, which is a common phenomenon in plant transformation experiments and likely results from biological factors such as gene silencing, the positional effects of transgene insertion, and individual plant differences. However, both CsCCD2L and CroCCD2 successfully facilitated the biosynthesis of crocin in tomato fruits, yielding comparable levels of crocins, although on average, the plants expressing CsCCD2L yielded tomatoes with a higher content of crocins [AVG = 2436.11 µg/mg] compared with plants expressing CroCCD2 [AVG = 982.78 µg/mg]. Specifically, the line MMO17.2 accumulated over 4.7 mg/g DW of crocins, whereas the line MMO16.7 produced approximately half that amount, highlighting the significantly enhanced performance of the CsCCD2L enzyme in the tomato fruits of MMO17.2. The biological implications of these differences warrant further consideration. Given that both alleles were expressed under the same regulatory elements with similar genetic backgrounds, the observed differences in crocin accumulation are unlikely to be due to expression level alone. Instead, they more plausibly reflect intrinsic differences in enzyme structure and catalytic efficiency. This aligns with the known diversity in CCD enzyme activity among species and suggests that even subtle sequence variations may significantly influence substrate binding or reaction kinetics.

When these results are compared to previous data obtained from CsCCD2L expression with the *Hp3/B^Sh^* background, which reached crocin levels of up to 6.5 mg/g DW [19], it becomes evident that the availability of zeaxanthin plays a critical role in determining crocin accumulation. The higher crocin content with the *hp3/B^Sh^* background likely reflects increased metabolic flux toward zeaxanthin, underscoring the importance of substrate availability for optimal CsCCD2L-mediated crocin biosynthesis.

## 5. Conclusions

Crocin is a bioactive apocarotenoid with notable therapeutic potential in the prevention and management of neurodegenerative disorders. Its incorporation into the human diet, either via traditional sources or through biofortified foods, offers a promising, non-pharmacological strategy for attenuating age-related neurological decline. However, natural sources of crocin are limited by low yield and cultivation challenges, necessitating alternative production systems. In this study, tomato was effectively utilized as a heterologous platform for crocin biosynthesis. Through targeted manipulation of the carotenoid pathway—specifically, the suppression of zeaxanthin epoxidase [ZEP] and the fruit-specific expression of CsCCD2L and CroCCD2—substantial crocin accumulation was achieved. Importantly, CsCCD2L showed higher catalytic efficiency than CroCCD2, despite comparable expression contexts. This suggests that the enzyme origin and inherent biochemical properties—rather than expression levels alone—play a key role in determining metabolic output. From a metabolic network perspective, ZEP suppression successfully re-routed carotenoid flux by increasing zeaxanthin availability, thereby enhancing substrate supply for CCD2-mediated cleavage. This demonstrates a fundamental synthetic biology principle: the combination of precursor pool enhancement and the careful selection of enzyme variants can synergistically redirect flux toward desirable end products. The integration of these strategies enabled efficient apocarotenoid production in a non-native host. Moving forward, future work should explore the stability of crocins under environmental stress conditions, evaluate broader nutritional impacts beyond crocin accumulation, and assess the long-term effects on fruit development and quality. These steps are essential to translate this metabolic engineering strategy into practical applications in the food and health sectors.

## Data Availability

The data supporting our findings are available in the manuscript file or from the corresponding author upon request.

## Supplementary Materials

The following supporting information can be downloaded at: https://www.mdpi.com/article/10.3390/biology14070850/s1, Figure S1: HPLC chromatograms of representative crocin profiles from MMO16.5 and MMO17.2 fruits. Inset shows the crocin spectra; Table S1: Oligonucleotides used for plasmid construction and expression analyses.
