# Supplementary material for: Natural CCD2 Variants and RNA Interference for Boosting Crocin Biosynthesis in Tomato
Source: Biology (Basel). 2025 Jul 12;14(7):850. doi: 10.3390/biology14070850 (PMC12292393; doi:10.3390/biology14070850)
Supplement: Supplementary file 1 [file biology-14-00850-s001.zip › biology-3715506-supplementary.pdf]

Figure S1

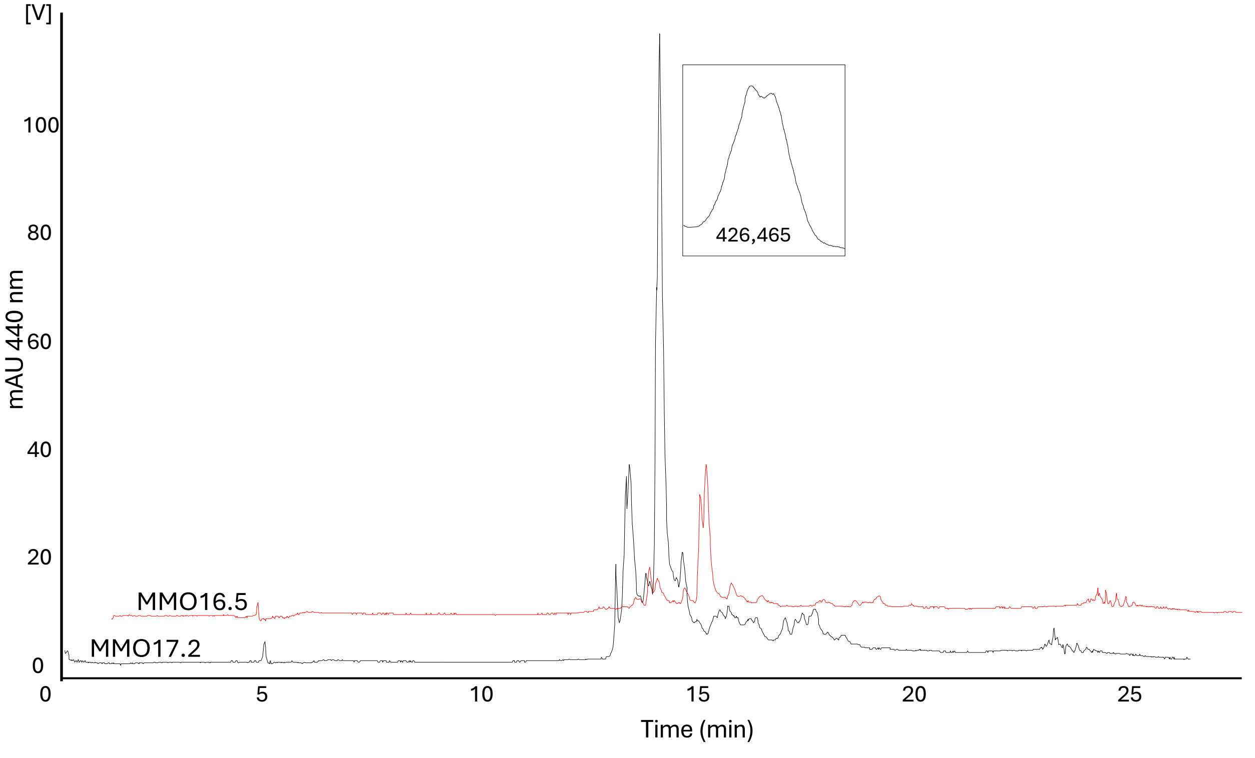

Figure S1. HPLC chromatograms of representative crocins profiles from MMO16.5 and MMO17.2 fruits. Inset is shown the crocins spectra.

Supplementary Table S1. Oligonucleotides used for plasmid construction and expression analyses.

rimers	Sequence 5´-3´
JPD2-Dom-CsCCD2-L-F	GCGCCGTCTCGCTCGAATGGAATCTCCTGCTACTAAAT
JPD2-Dom-CsCCD2-L-R	GCGCCGTCTCGCTCAAAGCTCATGTCTCTGCTTGGTG
JPD2CroCCD2F1outer	GCGCCGTCTCGCTCGAATGGATGCATCTAAACCTCTG
JPD2CrocCCD2R1inner	GCGCCGTCTCGAGATCTCCAATCTTCATAAATTTTGC
JPD2CroCCD2F2outer	GCGCCGTCTCGATCTAAGAGGATTGCTTGGATTG
JPD2CrocCCD2R2inner	GCGCCGTCTCGCTCAAAGCTCATGCCACTGCTTGCTG
epo-RNAi_F	GCGCCGTCTCGCTCGAATGCCGTCTTTGCATGATATCC
epo-RNAi_R	GCGCCGTCTCGCTCAAAGCTTATTAACCCACAAATCCC
JPD2-UGTgg-F	GCGCCGTCTCGCTCGAATGGAAGCTGGTGGTGATAAA
JPD2-UGTgg-R	GCGCCGTCTCGCTCAAAGCCTACCCTTTTGCTGTTCCA
sCCD2L-F	ACATGTCGCCTTGAGAGTCC
sCCD2L-R	TCAGATTTGATGCCAGGTTG
roCCD2-F	GTTGTCGCATCCACTCACTG
roCCD2-R	ATGGCAGGGGATGGAATTGA
CTIN2-F	CATTGTGCTCAGTGGTGGTTC
CTIN2-R	TCTGCTGGAAGGTGCTAAGTG
EP-F	CGTGCTGTTGGTGAGGAGAT
EP-R	AGTAGCTTCACTGGGTCCGA
